# Molecular Mechanisms of Neurogenic Inflammation of the Skin

**DOI:** 10.3390/ijms24055001

**Published:** 2023-03-05

**Authors:** Luiza Marek-Jozefowicz, Bogusław Nedoszytko, Małgorzata Grochocka, Michał A. Żmijewski, Rafał Czajkowski, Wiesław J. Cubała, Andrzej T. Slominski

**Affiliations:** 1 Department of Dermatology and Venerology, Faculty of Medicine, Collegium Medicum in Bydgoszcz, Nicolaus Copernicus University in Torun, 85-094 Bydgoszcz, Poland; 2 Department of Dermatology, Venereology and Allergology, Medical University of Gdansk, 80-210 Gdansk, Poland; 3 Molecular Laboratory, Invicta Fertility and Reproductive Centre, 81-740 Sopot, Poland; 4 Department of Histology, Faculty of Medicine, Medical University of Gdansk, 80-210 Gdansk, Poland; 5 Department of Psychiatry, Medical University of Gdansk, Debinki St. 7 Build. 25, 80-952 Gdansk, Poland; 6 Department of Dermatology, University of Alabama at Birmingham, 500 22nd Street South, Birmingham, AL 35294, USA; 7 Comprehensive Cancer Center, University of Alabama at Birmingham, 1824 6th Avenue, Birmingham, AL 35294, USA

**Keywords:** molecular mechanisms, neurogenic inflammation

## Abstract

The skin, including the hypodermis, is the largest body organ and is in constant contact with the environment. Neurogenic inflammation is the result of the activity of nerve endings and mediators (neuropeptides secreted by nerve endings in the development of the inflammatory reaction in the skin), as well as interactions with other cells such as keratinocytes, Langerhans cells, endothelial cells and mast cells. The activation of TRPV–ion channels results in an increase in calcitonin gene-related peptide (CGRP) and substance P, induces the release of other pro-inflammatory mediators and contributes to the maintenance of cutaneous neurogenic inflammation (CNI) in diseases such as psoriasis, atopic dermatitis, prurigo and rosacea. Immune cells present in the skin (mononuclear cells, dendritic cells and mast cells) also express TRPV1, and their activation directly affects their function. The activation of TRPV1 channels mediates communication between sensory nerve endings and skin immune cells, increasing the release of inflammatory mediators (cytokines and neuropeptides). Understanding the molecular mechanisms underlying the generation, activation and modulation of neuropeptide and neurotransmitter receptors in cutaneous cells can aid in the development of effective treatments for inflammatory skin disorders.

## 1. Introduction

The primary role of skin nerve endings is to sense and respond to external factors, as well as to provide the body with an organized means of protection from environmental threats [1]. Afferent fibers, unmyelinated C-fibers, myelin-type Aδ fibers and autonomic nerve fibers are present in the skin, and are characterized by a dense distribution throughout all of its layers. Neuropeptides are released from these fibers, and are also part of the cutaneous neuroendocrine system and stimulated by nociceptive stimuli [2,3]. The role of neuropeptides (neuromodulators, neurotransmitters and neurohormones) in the regulation of lympho- cells, mast cells and other cells of the immune system consists in the transduction of neurological impulses from afferent nerve fibers to signals that can be read by immunocompetent cells, and this carries the potential to exacerbate the inflammatory response [4,5,6,7]. The observation that various chronic inflammatory skin disorders, e.g., atopic dermatitis and psoriasis, are characterized by enhanced neurotrophin expression and peptidergic nerve fibers supports these pathophysiologic phenomena [8]. Various chronic inflammatory skin disorders, such as atopic dermatitis, prurigo nodularis, rosacea and psoriasis, exhibit increased expression of neurotrophins and the presence of nerve fibers that contain peptides. These observations provide evidence that these diseases share similar pathophysiological mechanisms. The neuropeptides released from nerve fibers can stimulate keratinocytes, which then trigger the release of proinflammatory cytokines such as IL-1α, IL-6 and IL-8 in the epidermis [2,9,10]. The epidermis undergoes close interaction with nerve endings, as well as the epidermis and nerves, thus producing factors for mutual communication. Secreted from the skin nerve endings, neuropeptides, such as SP (substance P) and CGRP, bind to receptors on the surface of mast cells and activate them, leading to degranulation and the release of many pro-inflammatory cytokines and vasoactive amines. SP, CGRP and VIP are powerful histamine releasers from mast cells, and function via an independent reaction with IgE bound to the surface of mast cells. Tachykinins directly cause dilatation and increase capillary permeability, which leads to edema and indirectly causes erythema by releasing histamine from mast cells. The mediators released during mast cell degranulation increase inflammation. Mrgprs (Mas-related G-coupled protein receptors), TRPA1 and PAR-2 (protease-activated receptor 2) [11,12] play a significant role in inducing itching and inflammation. When the MrgprX1 receptor is activated, it triggers the degranulation of mast cells. This, in turn, can result in the development of neurogenic inflammation through communication with cutaneous and sensory nerve cells [4]. As a result of this physiological process within the skin, mediators are released directly from the cutaneous nerves and initiate an inflammatory response, which leads to erythema, swelling and pain. 

Cutaneous neurogenic inflammation is a common element of chronic inflammatory skin disorders such as psoriasis, atopic dermatitis (AD), sensitive skin [13], rosacea [14], and hypertrophic scars [15].

## 2. Neuroimmune Communication (NIC)

The neuro-immuno-cutaneous (NIC) and neuro-immuno-cutaneous-endocrine (NICE) systems are based on a complex and ongoing communication network involving neuropeptides, cytokines, neurotransmitters, small molecules and other less defined factors, such as psychological stress [16]. These elements collaborate to maintain skin homeostasis, allowing the skin to detect and interpret environmental changes through the cutaneous neuroendocrine system, which uses chemical, physical and biological signals to regulate both local and global homeostasis. Neurogenic factors play an important role in the pathogenesis of skin inflammation, and there is a close relationship between the peripheral and central nervous systems, as well as between the endocrine and immune systems. The presence of numerous nerve cell endings in the skin, its rich vascularity, and the fact that it is the largest and most exposed organ to the action of harmful factors emphasizes its unique and important role in the pathogenesis and regulation local inflammation [1]. 

### 2.1. Neuropeptides (NPs)

The expression of receptors for neuropeptides (NPs), such as SP, has been found on endothelial cells, where after the activation of NK-1R receptors, endothelial cell proliferation and vascularization occur, increasing the expression of (VCAM)-1. ICAM-1 expression is increased both by the direct action of SP via NK-1R and by TNF-α, IL-1 and IFN-γ [4,17,18,19]. 

SP, together with CGRP released from peripheral nerve endings under the influence of a nociceptive stimulus, induces the translocation of P-selectin to the membranes of endothelial cells and the expression of E-selectin, intensifying inflammation. In addition, SP enhances the migration and endothelial adhesion of leukocytes and monocytes, and affects the vascular and cellular components of inflammation [6,20,21,22].

### 2.2. Neurotrophins (NTs)

The presence of neurotrophins (NTs) in the skin is crucial at every stage of the inflammatory response. Neuropeptides produced by cells present in the skin belong to three groups: opioid and non-opioid neuropeptides, and neurotrophins. Receptors for neurotrophins are found on keratinocytes, hair follicles, inflammatory cells such as T lymphocytes, macrophages, leukocytes and MCs. The most important neurotrophins produced in the skin include NT-3, NT-4, NGF (nerve growth factor) and BDNF (brain-derived neurotrophic factor). NGF is synthesized and released by keratinocytes, Merkel cells, fibroblasts and mast cells. NGF is the most important neurotrophic factor in dermal sensory nerves [6]. Skin diseases, which are clinically characterized by intense itching and histologically characterized by an increased number of nerve fibers in the skin, are regulated by neurotrophins [16,23,24].

#### 2.2.1. Calcitonin Gene-Related Peptide (CGRP)

The calcitonin gene-related peptide (CGRP) is one of the most prominent neuropeptides and is localized throughout the peripheral and central nervous system [25]. 

In thin unmyelinated sensory fibers in the dermal papillae, and in epidermal free nerve endings present in the dermal papillae, CGRP coexists with SP and causes pruritus. CGRP is also found in the perivascular region and is responsible for vasodilation in the skin without causing pruritus [26]. Neuroimmune reactions of the skin are bidirectional. The cutaneous nervous system can also be activated by cytokines released by immune cells [27]. The immune system takes note of pathogenic events through a set of receptors that recognize pathogen-associated molecular patterns (PAMPs), e.g., LPS and CpG, and damage-associated molecular patterns (DAMPs); some examples include high-mobility group box 1 (HMGB1), S100 proteins, and heat-shock proteins (HSPs) [28,29,30]. After binding with PAMPs or DAMPs, the described pattern-recognition receptors (PRRs), including Toll-like receptors (TLRs) and IL-1R, elicit inflammatory and immune responses through signaling to nuclear factor κB (NF-κB), thus inducing the expression of proinflammatory cytokines, for example, IL-1, -6, -31, IFN-γ (interferon-γ) and TNF-α (tumor necrosis factor-α) [31]. The cytokines that are released play the roles of ligands and activators of sensory nerves, and downstream neuronal effects take place. One example is IL-6, which triggers the expression of NGF and NT-3, 4 and 5; on the other hand, IL-31 exerts pruritic effects [32,33,34]. 

Melanocytes and sensory nerve endings interact via CGRP, which affects melanocytes by upregulating melanogenesis and increases melanocyte dendriticity by inducing keratinocyte-derived melanotropic factors [27]. CGRP affects the melanogenesis process when the skin is exposed to CGRP and when melanocytes are stimulated. During the melanogenesis process, the addition of CGRP-stimulated keratinocyte conditioned medium (CGRP-KCM) has been shown to stimulate melanogenesis; therefore, it is likely that keratinocytes produce melanotropic factors when stimulated with CGRP [6].

#### 2.2.2. Substance P (SP) and Its Role in Neuroinflammation

Substance P (SP) is a protein that consists of 10 amino acids. Together with neurokinin A and neurokinin B, it belongs to the family of neuropeptide tachykinins, whose wide range of activity is possible due to their presence in the nervous, digestive and immune systems. When substance P is released, it binds to its NK-1R receptors on various target cells. The linkages between neuropeptides and immune cells play an important role in the modulation of the inflammatory neurogenic process [35,36].

Substance P is released from the terminals of afferent unmyelinated C-fibers and myelin-type delta A-fibers in response to nociceptive stimulation [37], and plays a bigger part in itching than in pain [22]. It binds to keratinocytes or MCs [38], or induces the release of interleukins (as well as other cytokines) [39]. We can distinguish three main directional effects of SP activity: vasodilation, the activation of B lymphocytes and the increased proliferation of keratinocytes and fibroblasts [22,34,38,40,41]. SP plays an important role in AD, where the degranulation of mast cell granules leads to the release of proteases and histamine. The secondary mediators are leukotrienes and prostaglandins, while the substances secreted after the activation of MCs are interleukins such as IL-1, IL-2, IL-4, TNF-α and INF-γ [42,43]. At the level of the spinal cord, SP plays a role in pain neurotransmission and the modulation of autonomic stimuli. In the peripheral nervous system, SP receptors have been demonstrated on primary sensory neurons, where SP is regulated by nerve growth factor (NGF) [39]. SP initiates the degranulation of MCs, resulting in the release of activators of the inflammatory process and the hypervascularization and infiltration of mononuclear cells [34,38,41]. Individual neuropeptides/mediators and their roles in neuroinflammation are summarized in Table 1. 

## 3. Receptors in Neuro-Immune Interaction

### 3.1. Neurokinin Receptor (NK-R)

Cells that are resident and temporarily present in the skin express different types of receptor for neuropeptides. SP, NKA and NKB bind to the G protein-coupled receptors NK-1R, NK-2R and NK-3R, respectively [52]. When NK-1R is activated via SP, it causes multiple signaling cascades that involve the degranulation of mast cells and release of proinflammatory mediators; examples include histamine, and NGF expression and the production of leukotriene B4 (LTB4) in keratinocytes, leading to neurogenic inflammation and pruritus [53,54,55]. Several studies have researched the role played by SP and NK-1R in the mechanism of itching in various diseases such as atopic dermatitis, psoriasis and chronic idiopathic urticaria (CSU) [56,57]. 

It is suggested that NGF and its receptor TrkA play a major role in pruritus and allergic diseases. In an active process of inflammation, *NGF* expression is markedly upregulated in nerves related to the inflamed area, while increased levels of NGF are associated with skin dermatoses such as psoriasis [58]. The role of NGF consists in the maintenance, proliferation and growth of nerve cells. The process of cutaneous inflammation involves the NGF-dependent production of SP, CGRP and other neurotransmitters and neuropeptides or molecules linked with nociception. Furthermore, NGF has a direct stimulating effect on the degranulation of mast cells, enhancing the count of mast cells in peripheral tissues and favoring the growth of myeloid cells [59], thus promoting the survival of several immune cells in the cutaneous system, including eosinophils, monocytes, neutrophils, T cells and macrophages. NGF also induces the proliferation and differentiation of B cells and encourages the release of histamine from basophils. NGF can also stimulate IL-1 expression in PC12 cells and suppress the production of LTC4 in human eosinophils [52,60,61].

### 3.2. Tropomyosin Receptor Kinase A

The first information about TrK receptors appeared in 1986. A family of tyrosine kinase receptors, which include TrkA, TrkB and TrkC, was isolated. Trk receptors are stimulated by multiple neurotrophins, including NGF, BDNF, NT-3 and NT-4 [52,62]. Keratinocytes are the most important NGF exit point in the skin. In addition, NGF is produced by immune cells and neurons in a dynamic inflammatory process [63,64]. 

Increased concentrations of cytosolic Ca^2+^ induce the release of neuropeptides from the sensory nerves in the skin. There are five important GPCRs that play leading roles in neurogenic inflammation, including PAR-2 and PAR-4, and the Mas-related G-coupled protein receptors C11, A3 and X [65,66], in addition to the temporary receptor capacity of the vanilloid TRPV1 and the ankyrin TRPA1 [11].

### 3.3. Mas-Related G-Coupled Protein Receptors (Mrgprs)

In the Mrgprs family, we distinguish nine subgroups from MrgprA to MrgprH and MrgprX [67], which are characterized by low specificity for ligands and potentially high specificity for itching substances. This extensive group of receptors is characterized by low ligand specificity, and could have the best affinity for itch-inducing substances. Activation of the MrgprA3, C11 and X1 receptors is responsible for the peripheral itching sensation and scratching behavior. The receptors MrgprA3 and MrgprC11 are present on mast cells and on nerve endings, as well as on non-neuronal cells [68]. Activation of the Mrgpr receptor on mast cells causes itching and triggers strong scratching behavior, damaging the skin barrier, and thus, its immune homeostasis. When activated, MrgprX1 triggers the degranulation of mast cells and causes the engagement of sensory nerves and cutaneous cells in the development of neurogenic inflammation [69]. MrgprA3 and C11 are involved in the production of certain neuropeptides by sensitizing the TRPA1 and TRPV1 channels located on sensory nerve endings [70,71]. Mrgprs and the TRPA1 and PAR-2 receptors play a major role in itching and skin inflammation [4].

### 3.4. Transient Receptor Potential (TRP)

The temperature-sensitive channels, which are part of the transient receptor potential (TRP) superfamily, play a major part in the biology of the skin. Inflammatory processes within the skin are triggered by the activation of TRPV1 and TRPA1 receptors and result in the development of neurogenic inflammation in conditions such as psoriasis and AD [72,73,74,75]. The produced neuropeptides affect skin cells that increase the expression of similar neuropeptide receptors, including microvascular and MC cells, which results in vasodilation, degranulation and the release of plasma proteins and white blood cells [4,76]. The increase in the level of Ca^2+^ in the cytosol causes the exocytosis of neuropeptides and inhibits or stimulates the potency of several inflammatory genes that encode cytokines, neuropeptides and matrix metalloproteinases (MMPs), playing a leading role in dermatitis [77,78]. Cation channels containing the TRP receptor are involved in the exocytosis of neuropeptides responsible for the mechanism of neurogenic inflammation. The temperature-dependent nociceptive cation channel TRPV1 responds to high temperature (>43 °C) and its agonist capsaicin, which is found in chilies peppers [79]. The activation of TRPV1 and rapid Ca^2+^ influx release neuropeptides such as SP and CGRP and contribute to neurogenic inflammation. During cutaneous neuritis, the released neuropeptides and other mediators sensitize or activate TRPV1, leading to the maintenance of CNI [6,80]. TRPV1 is present in skin cells, such as keratinocytes, mast cells and dendritic cells, that act as pain sensors and chemical stimuli [81]. 

TRPA1 modulates the inflammatory response in keratinocytes by intensifying the potency of pro-inflammatory cytokines and prostaglandin E2 (PGE_2_) [82], which are involved in cutaneous inflammation and pruritus. They also activate the growth of HSP, which is responsible for the increase in pro-inflammatory cytokines in allergic skin diseases [83,84]. In conclusion, the activation of TRPA1 causes the production of several inflammatory mediators by keratinocytes.

TRP ion channels are involved in cutaneous thermosensation, osmoregulation and inflammation, as well as cellular growth. When pathological conditions such as inflammation or tissue injury are present, TRP is involved in signaling painful and pruritic stimuli to the CNS. Therefore, the identification of ion channels that detect heat or cold provides major insight into the molecular foundations of neurogenic inflammation, pain and pruritus. Furthermore, some TRPs (TRPV1 and TRPV4) appear to play a direct part in peripheral neurogenic inflammation [85,86,87]. TRPV1 and TRPA1 play a leading role in neurogenic dermatitis by means of the release of neuropeptides (including SP and CGRP) and pro-inflammatory cytokines [80,88].

### 3.5. Role of Protease-Activated Receptors (PARs)

TRPV1, TRPA1 and proteases PAR-2 and PAR 4 cause an increase in intracellular Ca^2+^ (iCa^2+^) concentration, the exocytosis of neuropeptides, and the expression of pro-inflammatory genes, leading to the development of CNI [78,89].

PARs are G protein-coupled receptors, of which there are four different subtypes: PAR1-4. They are so-called ‘‘alarm receptors’’ that do not possess classic ligands, but are activated by N-terminal proteolytic cleavage and by environmental proteases. The activation of PAR2 and PAR4 has been attributed to itching or pain in atopic dermatitis. *PAR2* is expressed by keratinocytes, ECs, MCs and sensory nerves. PAR 2 stimulation leads to the release of itch-inducing factors (e.g., ET-1, IL-33, TSLP and SP) from keratinocytes, EC, nerves and other neuroinflammatory mediators (TSLP and kallikrein) [81,90,91,92]. There are receptors for TSLP and PAR2 on nerve endings. Proteases activate the pathways of C-fiber excitation by binding to the PAR2 receptor. In AD, as a result of mast cell degranulation, tryptase is released and nerve endings are stimulated, which leads to histamine-independent pruritus [27].

Sensory neurons and keratinocytes harness the power of PAR2, which is stimulated by proteases triggered by degenerated MCs. In AD, the levels of tryptase and PAR2 are elevated in the patient’s skin, and the excessive secretion of PAR2 in keratinocytes is sufficient to cause AD-like changes [12,92]. AR-2 activation results in the stimulation of TRPV4 channels in dorsal root ganglion (DRG) neurons and of the NF-κB pathway in keratinocytes [93,94]. After the secretion of proteases by MCs, activated PAR2 stimulates endogenous inflammation, itching and pain, which are dependent on CGRP and substance P in AD patients. In an addition, exogenous factors such as allergens can activate PAR-2, thereby contributing to itching and pain [88,95]. TRPV1, TRPA1, and PAR-2 and PAR-4 proteases are also present in cells that reside in and infiltrate the skin during CNI, which enhances skin signaling and may exacerbate AD [96].

## 4. Mast Cells as Major Mediators of Neuroimmune Crosstalk

### 4.1. The Role of Mast Cells in Neuroinflammation

Mast cells (MCs) are resident immune cells in the skin. They are produced in the bone marrow from CD34+ cells and are the "first responders" of the immune system in the skin. They affect every stage of the immune response, especially the initial stage, and are often referred to as “gatekeepers” [97] because they are in the immediate vicinity of blood vessels and nerve endings.

Mast cells gather near the afferent innervation of the periphery, visceral organs, and the meninges [98,99]. Furthermore, mast cells release numerous different mediators, which initiate nociception in primary afferent neurons by binding to the corresponding receptors [100,101]. Mast cells are usually located within the dermis, and they are also present near the nerve endings in the epidermis. Mast cells have also been detected in the epidermis in psoriasis, epidermal hyperplasia and chronic inflammation [101].

Mast cells are also found in the central nervous system; they are located on the inside of the blood–brain barrier and affect its permeability [102,103]. Stress and comorbidities are the main factors responsible for their presence and activity [104]. Mast cells can react with brain cells, leading to an increase in the influx of pro-inflammatory cytokines and contributing to the exacerbation of neuroinflammation [98].

### 4.2. Mast Cell-Induced Disease

Mast Cell Activation Diseases (MCADs) are MC-associated inflammatory conditions, i.e., mastocytosis and MC activation syndrome (MCAS) [105,106,107,108,109,110,111]. Mastocytosis is a unique disease expressed by a D816V mutation in the c-KIT gene, leading to the formation of abnormal mast cells in the skin or internal organs. Compared to MCAD, MCAS is a more common non-proliferative disorder with increased MA stimulation. Ailments have a wide range and intensity. However, pain and pruritus are among the most common symptoms, varying in severity and localization in MCAS and mastocytosis [101].

**Figure 1:**
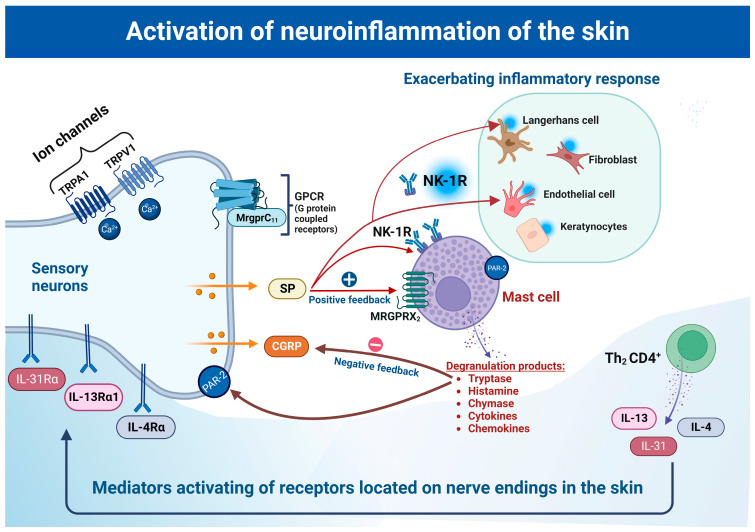
In the neurogenic inflammatory pathway, peripheral nerve endings communicate with various skin cells, such as keratinocytes, melanocytes, fibroblasts and MCs, and also with immune cells via neurotrophins and neuropeptides. In neurogenic inflammation, an important role is played by MCs, on the surface of which there are numerous receptors for neuropeptides secreted by nerve endings. After activation of the receptors, mast cells degranulate and release proteases, cytokines and histamine. Tryptase binds to the PAR-2 receptor, activating it and releasing neuropeptides such as CGRP and SP, which are responsible for itching and scratching. PAR-2 activation is associated with pain perception. Tryptase directly affects CGRP, causing its degradation and negative feedback. On the other hand, SP, acting on the NK-1R and MRGPRX2 receptors on mast cells, activates them, causes cell degranulation and intensifies the inflammatory response. This response also involves TH2CD4+ immune cells that release cytokines IL-4, IL-13 and IL-31; these cytokines, as mediators, activate receptors on nerve endings, which intensifies itching in skin diseases. Ca^2+^-dependent TRPV1 and TRPA1 ion channels can communicate with each other and, when activated, increase the release of neuropeptides, thereby exacerbating neurogenic skin inflammation.

Mast cells have receptors on their surface that allow them to communicate with their environment. The high-affinity IgE receptor FcεRI plays an essential role in the release of histamine, tryptase, leukotrienes and prostaglandins, which results in local vasodilation, edema, local neurogenic stimulation and inflammatory cell infiltration [112,113,114]. The degranulation of mast cells and the release of tryptase leads to the production of nociception through the binding of protease-activated receptor 2 (PAR-2) to primary afferents. PAR-2 is part of a G protein-coupled receptor expressed on primary sensory neurons. Peripheral nerve endings release P and CGRP after the activation of PAR-2 (Figure 1) [91,115].

Mast cells are responsible for releasing histamine, which acts through its four receptors. When activated, the H1 receptor leads to hypotension and edema. H3 is expressed in the CNS, and H4 on granulocytes and mast cells. The G protein-coupled MRGPRX2 receptor (Mas-related G protein-coupled receptor X2) comprises a human analogue of the murine Mrgprb2 [116,117].

In a study by Meixiong et al., which used a knockout mouse model, it was proven that deficiency of the Mrgprb2 receptor reduces skin pruritus in allergic contact dermatitis. Activation of the Mrgprb2 receptor causes non-histaminergic itching. The results of this study indicate the possibility of a new therapeutic route using Mrgprb2 [46,118].

The bipolar connection between the MC and nerves has been proven to codify itching, pain and inflammatory responses. MRGPRX2 is stimulated by neuropeptides (e.g., SP) released from sensory neurons [98,112,118]. In mice, innate immune cells recruited via SP/Mrgprb2 receptor interaction to the site of inflammation trigger neural inflammation linked to pain and itching responses [116,118]. In mice, it is suggested that tryptase B2 could be a major mediator secreted by MCs, activating sensory neurons by means of receptors stimulated by protease (PAR1, PAR-2, PAR-4). These receptors on neurons are highly expressed and are responsible for itching and scratching [46,47].

## 5. Influence of Stress in Formation of Neurogenic Skin Inflammation

**Figure 2:**
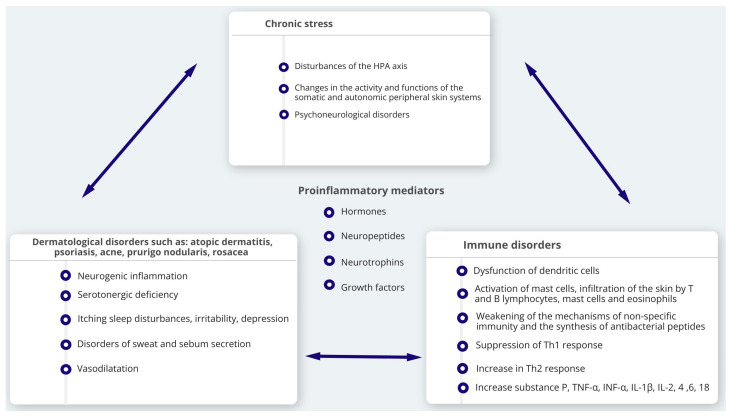
The role of stress and mediators produced by cells of the nervous system, immune system and resident cells of the skin in the induction and exacerbation of symptoms of chronic dermatitis and the formation of disorders of specific and non-specific immunity of the skin.

Mental stress is a state in which a person feels strong emotions such as anxiety, fear, helplessness, anger and aggression and cannot control them. As a result, numerous biochemical and physiological changes can disturb homeostasis and disrupt mental and physical balance, which involves the immune and endocrine systems. Stress can induce and exacerbate various skin conditions, from cancer to inflammatory diseases such as psoriasis, acne, AD, CSU, seborrheic eczema, lichen planus and alopecia areata [48,119]. The effects of stress on the skin can be direct (via the peripheral nervous system) or indirect (via the endocrine and immune systems) [3,120,121,122]. Neurogenic inflammation is the result of the participation of nerves and their mediators, neuropeptides. Prior research has demonstrated the effect of stress on the processes involved in the cutaneous neurogenic reaction, such as altering the density and activity of nociceptive nerve fibers that are vulnerable to capsaicin, altering the activity of their neurons, enhancing the level of SP release from the unmyelinated nerve endings of the skin and degranulating cutaneous mast cells. Neuroinflammatory processes are responsible for the development of depression [123]. The activation of meningeal cell receptors that results from these processes to a low degree, and the activation of immune cells, are promoted by the endogenous process of depression (Figure 2) [124,125].

**Figure 3:**
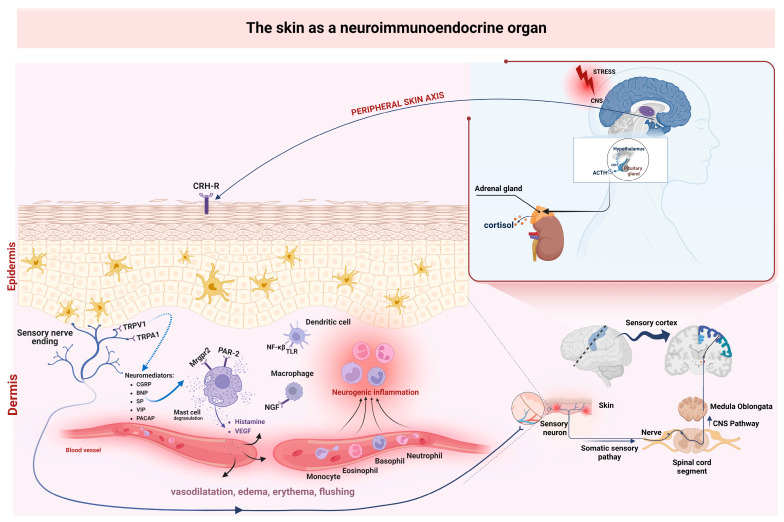
The central and peripheral axes of the HPA are in charge of proper skin barrier function and inflammatory reactions. The release of neuromediators (CRF) from the hypothalamus and other areas of the CNS that can stimulate the release of norepinephrine and cortisol from adrenal HPA, and the release of leukocytes in the circulatory system via CRF and MC receptors, which modulate immune responses during inflammation and immunity, are activated by various stressors. Inflammatory responses in the skin are modulated by cytokines and neuropeptides of immune cells. Protease-activated receptor 2 (PAR2) on the plasma membrane of sensory nerve endings is activated by tryptase from degranulated MCs, which, in turn, stimulates the release of calcitonin gene-related peptide and tachykinins from sensory nerve endings. Vasoactive sensory nerve peptides are released under the influence of mediators from mast cells and other inflammatory cells. The mobilization of intracellular Ca^2+^ by PAR -2 at the level of the spinal cord induces the secretion of CGRP and SP from the central nerve endings. Neuromediators that are released when stimulated by sensory nerves modulate cutaneous inflammation, pain and itching.

In response to stress, the hypothalamus, through the corticotropin-releasing hormone (CRH), stimulates the release of ACTH, activating the production of cortisol by the adrenal cortex [120,126]. The body’s primary line of defense in the face of environmental factors is the skin, and the second is molecular mechanisms, through which the body responds to threats and restores local homeostasis. The fundamental bodily system involved in the response to stress is the hypothalamic–pituitary–adrenal (HPA) axis. The presence of an analog of the HPA axis has been demonstrated in the skin, which is activated by various environmental factors [2,127,128]. Several hormonal changes occur due to stress factors in the HPA axis, which initiates CRF secretion in the hypothalamus; this hormone binds to a type 1 receptor in the anterior pituitary gland and stimulates the secretion of ACTH by the anterior pituitary gland through corticotropin-releasing hormone (CRH) production and secretion of the adrenocorticotropic hormone (ACTH) from POMC. ACTH travels through the bloodstream to the cells of the adrenal cortex and affects the production of glucocorticoids, to which cortisol—the so-called stress hormone—belongs. GC, through negative feedback, affects hormones secreted by the hypothalamus (CRF) and pituitary gland (POMC) (Figure 3) [129].

Glucocorticoids impact the organs via two types of receptor: mineralocorticoid (MR) and glucocorticoid (GR), which have different affinities and localization [130]. These receptors are mainly present in the hippocampus, the paraventricular nuclei of the hypothalamus and the pituitary gland. The binding of cortisol to MR and GR in the brain primarily suppresses the secretion of corticotropin-releasing hormone in the hypothalamus and adrenocorticotropic hormone in the pituitary gland. The regulation of the hypothalamic–pituitary–adrenal system works based on feedback, whereby glucocorticoids use glucocorticoid receptors to block the activity of the HPA axis at the level of the pituitary gland or hypothalamus, or indirectly by means of receptors within the hippocampus. Extended chronic stress disturbs the HPA axis and leads to the production of steroid hormones, which can impair a number of physiological processes, including neurogenesis and hormonal regulation. We observe enhanced adrenal responsiveness to ACTH in depressed patients [131]. 

As a response to cutaneous stress, KCs releases hormonal products, which are similar to those produced in systemic stressful events such as CRF, POMC, β-END, ACTH and α-MSH [1,2,121,132]. The enzymes CYP11A1, CYP21A2, 3β-HSD and CYP11B1, which are involved in the production of corticosteroids, are expressed in KCs [34,121,127,133,134,135,136]. Glucocorticoids are also produced by melanocytes [137], fibroblasts [138] and immune cells [139,140].

MCs can be activated by stress with the help of chemokines, neuropeptide cytokines and hormones. The presence of MCs around blood vessels close to neurons and microglia (meninges, thalamus and hypothalamus) allows them to be stimulated by CRF secreted from the hypothalamus in response to stress; this, together with neurotensin (NT), can stimulate MCs to release inflammatory and neurotoxic mediators that impair the blood–brain barrier (BBB), causing focal inflammation. The stimulation of MCs by CRF and NT increases vascular permeability and, through the induction of surface receptors on MCs, leads to paracrine and autocrine reactions. These reactions take place in the brain, especially in the cerebellum and diencephalon [141].

MCs that are present near microglial cells in the brain also produce CRH, which regulates other immune cells. The production of IL-1 family inflammatory factors is regulated by CRH, and they are released by MC, leading to an autocrine effect [142]. Nuclear factor κB (NF-κB) and activating protein-1 (AP-1) stimulate inflammatory MCs, which leads to the release of IL-33, TNF, IL-6, IL-5, IL-4, IL-1, IL-13 and GM-CSF, and various chemokines, including MIP-1α, MIP-1β and MCP-1 [143]. Neurotoxicity causes NO release by astrocytes under the influence of TNF from mast cells and other cytokines [144,145]. Brain inflammation is a major factor in the pathogenesis of neuropsychiatric disorders.

## 6. Conclusions

Neurogenic skin inflammation involves the activation and modulation of signaling pathways, which leads to the development of acute and chronic dermatological diseases such as psoriasis, atopic dermatitis or eczema. Investigating the mechanisms of CNI is of therapeutic importance, as individual receptors may be molecular targets for new drugs; the MRGPRX2 receptor, which plays a role in pruritus, seems to be particularly important. The clinical significance of the TRPV1 and TRPA1 channels in pruritus and pain is only partially understood. Targeting one or more TRP channel sensitization signaling pathways could open up new avenues for treating skin diseases. Protein signaling inhibitors may represent a new direction in the treatment of neurogenic dermatitis. Immune cells (dendritic cells, eosinophils and T lymphocytes) that are involved in the transmission of itching and the inflammatory response regulate communication by neurons. Peripheral nerves can communicate with other cells in the skin through alternative signaling pathways (cytokines, neurotrophins and neuropeptides). The proper functioning of the skin’s neurogenic system ensures the maintenance of systemic homeostasis, while dysregulation of the skin’s neuroendocrine system is associated with numerous diseases. Understanding all the components of this system may contribute improving the current understanding of the mechanisms and targeted treatment of skin diseases. 

## Figures and Tables

**Table 1:** Selected neuropeptides/mediators and their roles in neuroinflammation.

Neuropeptide/ Mediator	Sources/Expressed by	Cell types Expressing the Receptor	Overall Function in Skin	Association with Skin Diseases	Ref.
**SP (substance P)**	Secreted by sensory C fibers and DRG - Endothelial cells, keratinocytes and MCs	- NK-1R (neurokinin-1 receptors) - Endothelial cells, connective tissue mast cells producing tryptase and chymase, fibroblasts and Langerhans cells	- Initiates the inflammatory response, leading to proliferation of specific T-lymphocytes, as well as the activation and degranulation of mast cells in early stages of psoriasis - Vasodilatation - Local inflammation - Increased cellular proliferation - Skin HPA axis activation	- Psoriasis - Rosacea - Atopic dermatitis - High concentration is observed in the blood of patients, which correlates with the activity of the disease process and the intensity of itching	[4,17,18,19,20,21,22,35,36]
**NGF (nerve growth factor) neurotrophin**	Secreted by cutaneous cells (keratinocytes, fibroblasts and adipocytes nerves)	NGF-TrkA keratinocytes, MCs, fibroblasts and eosinophils	- Correlated with the intensity of pruritus - Modulates nerve innervation and neuropeptide release, degranulates mast cells and induces keratinocyte hyperproliferation - Responsible for proliferation and growth of nerve cells - Favors the survival of certain immune cells in the cutaneous system	- Psoriasis - Atopic dermatitis - Allergic diseases - Prurigo nodularis	[6,16,23,24]
**CGRP (calcitonin gene-related peptide)**	- One of the main peptides involved in neurogenic inflammation - Is released after the activation of TRPV1 or TRPA1 - Secreted by sensory neurons	CGRP receptors and mast cell surface	- Induces vasodilatation - Can activate eosinophils to release proinflammatory mediators - Stimulates the proliferation of keratinocytes - Is responsible for the appearance of erythema	- Psoriasis - Prurigo nodularis - Atopic dermatitis - Rosacea	[18,25,26,32,33,34,44]
**VIP (vasoactive intestinal peptide)**	Secreted by: - Sensory and autonomic neurons - Keratinocytes, endothelial cells and T lymphocytes	Membrane receptors VPAC1 and 2R coupled with G protein are found on keratinocytes, T lymphocytes and mast cells	- MC degranulation and the production of proinflammatory cytokines - Induces vasodilatation	- Eczema - Psoriasis - Atopic dermatitis	[18,43,45]
**CRF (corticotropin-** **releasing factor)** **the central neuropeptide of the HPA axis**	- Keratinocytes, melanocytes, fibroblasts and mast cells	- CRF1 receptors are found on the cells of the epidermis (keratinocytes and melanocytes) and the dermis (fibroblasts and mast cells) - CRF2 receptors are found on the cells of skin appendages (hair follicles, sebaceous and sweat glands)	- Stimulates the production and release of MCs, keratinocytes and fibroblasts of cytokines with a Th2 profile	- Psoriasis - Acne - Alopecia areata - Atopic dermatitis - Vitiligo - Lichen planus - Seborrheic dermatitis - Rosacea - Urticaria	[46,47,48,49]
**Substances that inhibit the neurogenic inflammatory process**					
**SST (somatostatin)**	- Receptors (SSTR2) are found on T lymphocytes, Langerhans cells, epithelium cells and fibroblasts	- Sensory neurons - Merkel cells - Langerhans cells - Sweat gland cells	- Inhibits the secretion of pro-inflammatory cytokines - Inhibits the secretion endocrine glands - Inhibits intracellular cAMP		[18,19,50]
**(α-MSH)—** **α-melanocyte stimulating hormone**	MC1R−MC5R melanocytes, keratinocytes, fibroblasts and immune cells	- Is formed on the skin by the POMC prohormone - Melanocytes, keratinocytes, MCs, macrophages and monocytes	- Inhibits the synthesis of cytokines with anti-inflammatory properties		[18,51]

## Data Availability

Not applicable.

## Abbreviations

α-MSH	α-melanocyte stimulating hormone
AD	atopic dermatitis
AP-1	activating protein-1
BDNF	brain-derived neurotrophic factor
CGRP	Calcitonin gene-related peptide
CGRP-KCM	CGRP-stimulated keratinocytes
CNI	cutaneous neurogenic inflammation
CRF/-H	corticotropin-releasing factor/- hormone
CSU	chronic spontaneous urticaria
DRG	dorsal root ganglion
DAMPs	damage-associated molecular patterns
ICAM	intracellular adhesion molecule
iCa^2+^	intracellular Ca^2+^
HMGB1	high mobility group box 1
HPA	hypothalamic-pituitary-adrenal
HSP	heat shock protein
MCAS	mast cell activation syndrome
MCs	mast cells
Mrgprs	Mas-related G-coupled protein receptors
MRGPRX2	Mas-related G protein-coupled receptor X2
MMPs	matrix metalloproteinases
NF-κB	nuclear factor κB
NGF	nerve growth factor
NGF-TrkA	nerve growth factor (NGF)-Tropomyosin-related kinase A receptor (TrkA)
NIC	neuro-immuno-cutaneous
NICE	neuro-immuno-cutaneous-endocrine
NK1- 3R	neurokinin-1−3 receptors
NPY	neuropeptide Y
NT-3/4	Neurotrophin-3/-4
PAF	platelet-activating factor
PAMP	pathogen-associated molecular pattern
PAR	protease-activated receptor
PG	prostaglandin
POMC	proopiomelanocortin
PRR	pattern-recognition receptor
SM	systemic mastocytosis
SP	substance P
SST	Somatostatin
TLR	Toll-like receptors
TrkA	tropomyosin receptor kinase A
TRP	transient receptor potential
TRPA	transient receptor potential Ankyrin
TRPV	transient receptor potential vanilloid
TSLP	thymic stromal lymphopoietin
VCAM	vascular cell adhesion molecule
VEGF	vascular endothelial growth factor
VIP	vasoactive intestinal peptide
